# Markov chains improve the significance computation of overlapping genome annotations

**DOI:** 10.1093/bioinformatics/btac255

**Published:** 2022-06-27

**Authors:** Askar Gafurov, Broňa Brejová, Paul Medvedev

**Affiliations:** Department of Computer Science, Comenius University, Bratislava 84248, Slovakia; Department of Computer Science, Comenius University, Bratislava 84248, Slovakia; Department of Computer Science and Engineering, The Pennsylvania State University, University Park, PA 16802, USA; Department of Biochemistry and Molecular Biology, The Pennsylvania State University, University Park, PA 16802, USA; Huck Institutes of the Life Sciences, The Pennsylvania State University, University Park, PA 16802, USA

## Abstract

**Motivation:**

Genome annotations are a common way to represent genomic features such as genes, regulatory elements or epigenetic modifications. The amount of overlap between two annotations is often used to ascertain if there is an underlying biological connection between them. In order to distinguish between true biological association and overlap by pure chance, a robust measure of significance is required. One common way to do this is to determine if the number of intervals in the reference annotation that intersect the query annotation is statistically significant. However, currently employed statistical frameworks are often either inefficient or inaccurate when computing *P*-values on the scale of the whole human genome.

**Results:**

We show that finding the *P*-values under the typically used ‘gold’ null hypothesis is NP-hard. This motivates us to reformulate the null hypothesis using Markov chains. To be able to measure the fidelity of our Markovian null hypothesis, we develop a fast direct sampling algorithm to estimate the *P*-value under the gold null hypothesis. We then present an open-source software tool MCDP that computes the *P*-values under the Markovian null hypothesis in $O(m^{2}+n)$ time and O(m) memory, where *m* and *n* are the numbers of intervals in the reference and query annotations, respectively. Notably, MCDP runtime and memory usage are independent from the genome length, allowing it to outperform previous approaches in runtime and memory usage by orders of magnitude on human genome annotations, while maintaining the same level of accuracy.

**Availability and implementation:**

The software is available at https://github.com/fmfi-compbio/mc-overlaps. All data for reproducibility are available at https://github.com/fmfi-compbio/mc-overlaps-reproducibility.

**Supplementary information:**

Supplementary data are available at *Bioinformatics* online.

## 1 Introduction

A genome annotation is a fundamental component of biological analysis. Mathematically, it can be viewed as a set of genomic intervals with some given characteristic. For example, it is used to represent repeat elements (Jurka, 2000), genes (Venter *et al.*, 2001) or non-coding RNAs (Bartel, 2009). Genome annotations are often compared against each other to determine whether the underlying processes generating them are related; this is sometimes referred to as *colocalization analysis*. For example, if one annotation contains the nucleosomes with H3K4Me3 histone modifications and another contains known promoter regions, then we can count the nucleosome regions that overlap at least one promoter region. A large number of overlapping regions would suggest that transcription initiation is correlated with the presence of H3K4Me3 histones (Guenther *et al.*, 2007). A robust measure of statistical significance becomes important in order to ascertain whether the overlaps are due to chance or due to a true association.

There have been several approaches to colocolization analysis, surveyed by Dozmorov (2017) and Kanduri *et al.* (2019). Kanduri *et al.* (2019) characterize these methods by (i) the choice of the colocalization statistic, e.g. the number of overlapping intervals (Layer *et al.*, 2013), the number of shared bases (Zarrei *et al.*, 2015) or the distances between the closest elements (Chikina and Troyanskaya, 2012), (ii) the choice of the null hypothesis, e.g. permutational (Coarfa *et al.*, 2014) or with fixed interval positions (Sheffield and Bock, 2016) and (iii) the algorithm to compute the *P*-value, e.g. an analytical (McLean *et al.*, 2010) or a sampling (Yu *et al.*, 2015) algorithm.

A natural and popular choice of the null hypothesis (which we call the *gold standard*, or $H_{0}^{GS}$) is that one of the two compared annotations (called *query*) is drawn uniformly at random from the set of all possible non-overlapping rearrangements of the query’s intervals, whereas the other annotation (called *reference*) is fixed. The gold standard and its extensions take into account the lengths of intervals and are more flexible than approaches such as those that fix interval positions (Kanduri *et al.*, 2019). However, null hypotheses of such type were, until recently, only used with *P*-value algorithms based on rejection sampling (Kanduri *et al.*, 2019). The drawbacks of rejection sampling are that it cannot accurately estimate small *P*-values and is too slow when the query has high coverage or a high number of intervals. A major advance was recently made by Sarmashghi and Bafna (2019), who gave two analytical (non-sampling) *P*-value algorithms under slight modifications to $H_{0}^{GS}$, showing a potential way to reap the benefits of $H_{0}^{GS}$ while overcoming its computational challenges.

The most promising algorithm of Sarmashghi and Bafna (2019), which we refer to as SBDP, assumes a modified $H_{0}^{GS}$ which is forced to maintain the order of intervals in the query. SBDP computes the exact *P*-value under this modified $H_{0}^{GS}$ in time O(νmnL) and memory O(νmL), where *m* and *n* are the numbers of intervals in the reference and query, respectively, *L* is the length of the chromosome, and 0<ν≤1 is a scaling factor. The scaling factor trades off speed/memory for accuracy by shrinking the chromosome and all the intervals by a factor of *ν*. Experiments show that SBDP tends to be accurate with respect to $H_{0}^{GS}$ when *ν* = 1, in spite of the order restriction. However, the running time is proportional to *L*, making SBDP only pseudo-polynomial and requiring substantial scaling to run on human annotations. Such scaling can have a detrimental affect on the accuracy and result in biased *P*-values (Sarmashghi and Bafna, 2019). Overall, though SBDP has for the first time allowed analytically computing *P*-values under $H_{0}^{GS}$ in many smaller instances, a method that is fast and accurate for the human genome has remained elusive.

In this article, we first show that calculating the *P*-value under $H_{0}^{GS}$ is NP-hard (Section 3), which explains the lack of algorithms that are both efficient and accurate and motivates the need for alternate null hypotheses. In order to test the extent to which alternate null hypotheses differ from $H_{0}^{GS}$, we give an algorithm for sampling under $H_{0}^{GS}$ in O(n log L) time per sample (Section 4). We are not aware of previous descriptions of a non-rejection-based sampling scheme. Then, we propose an alternate null hypothesis based on Markov chains (Section 5). Our Markov chain null hypothesis ($H_{0}^{MC}$) is that the query is generated by a Markov chain with two states, corresponding to ‘outside’ and ‘inside’ an interval, with transition probabilities that maximize the likelihood of observing the query *Q*. Under $H_{0}^{MC}$, the asymptotically expected lengths of the intervals and the gaps between them are equal to average lengths of the intervals and gaps in *Q*, respectively. We then give an algorithm MCDP to compute the exact *P*-value in time $O(m^{2}+n)$ and memory O(m) (Section 6), which avoids any dependence on the genome length *L*.

Our experimental results on both synthetic and real datasets show that MCDP is generally accurate with respect to $H_{0}^{GS}$ while using negligible memory and running efficiently on human-scale datasets (Section 7). MCDP outperforms SBDP in runtime and memory usage by orders of magnitude on human genome annotations, while maintaining the same level of accuracy. In our largest test case, we compared the set of all human exons (217 527 intervals) with the set of all known copy number losses (23 438 intervals), which is a nearly 10-fold increase in size in comparison to *H3K4me3*, the largest evaluation dataset of Sarmashghi and Bafna (2019). MCDP ran in <17 h and used less than half a gigabyte of RAM, while SBDP exceeded the memory capacity of our server (∼100 GB) when used with $\nu =10^{-3}$. Furthermore, we believe that the flexibility and power of our Markov chain approach allow modifications to the gold standard null hypothesis that address many of the biological use-cases described by Kanduri *et al.* (2019).

## 2 Preliminaries

Let *L* > 0 be an integer which we refer to as the *genome length*. Given two positions x∈{0,…,L} and y∈{0,…,L}, with *y* > *x*, the *interval* [x,y) refers to the interval between *x* and *y* – 1, including the endpoints. This type of interval is sometimes said to be *half-open* and to be contained in [0,L). The *length* of an interval is *y* – *x*. The *weight* of a set of intervals is the sum of their lengths. Let *A* be a set of non-intersecting intervals contained in [0,L). We let *lens*(*A*) denote the multiset of lengths of intervals in *A*. We let *gaps*(*A*) denote the sequence of lengths of the gaps between adjacent intervals of *A*, starting from the beginning and up to the end of the genome. For example, if *L* = 10 and A={[3,4),[5,6),[7,9)}, then lens(A)={1,1,2} and gaps(A)=(3,1,1,1). Given two sets of intervals *A* and A′, we let K(A,A′) denote the number of intervals in *A* that intersect some interval in A′. We say that a non-intersecting set of intervals is *separated* if all the gaps, except the first and the last, are at least 1. We formally define an *annotation* to be a set of non-intersecting intervals that is separated and contained in [0,L).

## 3 The *P*-value problem and the gold standard null

In this section, we define the *P*-value problem and the gold standard null hypothesis ($H_{0}^{GS}$) and then show that the *P*-value problem under $H_{0}^{GS}$ is NP-hard. Let *R* (reference) and *Q* (query) be two annotations. We assume that *R* is sorted. Let $H_{0}$ be a null hypothesis for the distribution of *Q*. The *P-value problem for the overlap between genome annotations* is to output the sequence $\left(p_{0},\ldots ,p_{\left|R\right|}\right)$, where, for 0≤k≤|R|,

$$p_{k}={\text{Pr}}_{Q_{\text{rand}}∼H_{0}}[K(R,Q_{\text{rand}})\geq k].$$

The *gold standard null hypothesis* ($H_{0}^{GS}$) generates an annotation $Q_{\text{rand}}$ uniformly at random from a sample space of all annotations such that $\mathrm{lens}(Q_{\text{rand}})=\mathrm{lens}(Q)$. We now show that the problem of computing *P*-values for the overlap between genome annotations under the gold standard null hypothesis is NP-hard. This helps explain the lack of a polynomial time algorithm and motivates us to consider an alternate null hypothesis.

Theorem 1. *The* P*-value problem for the overlap between genome annotations under the gold standard null hypothesis is* NP*-hard.*

Proof. We proceed by a reduction from the NP-complete *multiprocessor scheduling* problem (Garey and Johnson, 1979). The multiprocessor scheduling problem takes a multiset of *N* tasks with positive integer lengths $\vec{a}=\{a_{1},\ldots ,a_{N}\}$ and positive integers *w* and b≥2 and decides whether it is possible to partition the tasks into *b* (some possibly empty) batches, such that the sum of lengths for each batch is less or equal to *w*.

Let $\vec{a}$, *b* and *w* be an instance of the multiprocessor scheduling problem. Without loss of generality, we assume that all lengths in $\vec{a}$ are at least 2. This can be ensured by multiplying *w* and all input lengths by 2. Furthermore, without loss of generality, we assume that $wb\geq \sum_{i}a_{i}$, since otherwise the scheduling problem trivially has no solution.

**Fig. 1.**
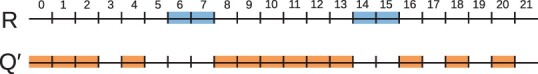
An example illustrating the reduction in Theorem 1 from an instance of the multiprocessor scheduling problem to an instance of the *P*-value problem under $H_{0}^{GS}$. The scheduling instance has $\vec{a}=\{2,2,2,2,4,7\}$, *N* = 6, *b* = 3 and *w* = 7. The resulting instance of the *P*-value problem has the length of the genome as *L* = 22, the reference interval set as R={[6,8),[14,16)}, and the multiset of query interval lengths as lens(Q)={1,1,1,1,3,6}. The figure shows *R* and an annotation Q′ with lens(Q′)=lens(Q) and no overlap with the reference (i.e. K(R,Q′)=0). The annotation Q′ corresponds to partitioning $\vec{a}$ as {{4,2},{7},{2,2,2}}, which is a valid solution to the scheduling problem instance

From this instance of the scheduling problem, we define an instance of the *P*-value problem under the $H_{0}^{GS}$. We set the genome length to be L:=b(w+1)−2 and define the reference annotation as having *b* – 1 intervals with interval *i* being [i(w+1)−2,i(w+1)), for 1≤i≤b−1. Conceptually, this partitions the genome into *b* equally sized *empty regions*, separated by *b* – 1 *reference intervals*. The length of each empty region is *w* – 1 and of each reference interval is 2. The query annotation *Q* is defined as *N* intervals whose lengths are given by subtracting 1 from each of the task lengths, i.e. $\mathrm{lens}(Q)=\{a_{1}-1,\ldots ,a_{N}-1\}$. Note that the locations of these intervals is irrelevant for the *P*-value problem under $H_{0}^{GS}$. Figure 1 depicts an example of the construction.

We claim that for any annotation Q′ with lens(Q′)=lens(Q), there exists a solution to the scheduling problem iff K(R,Q′)=0. For the if direction, suppose the elements of $\vec{a}$ are partitioned into *b* batches with the sum of lengths in each batch at most *w*. If we take all the intervals corresponding to a batch of tasks and place them side by side, with gaps of one between them, their total span on the genome will be at most *w* – 1. We can then define Q′ so that each of the *b* batches fits into one of the *b* empty regions, thereby assuring that K(R,Q′)=0. For the only if direction, assume that there exists a Q′ with K(R,Q′)=0. If an empty region contains *x* intervals, then, due to the gap of at least one between adjacent intervals, the sum of their lengths is at most *w* – *x*. Given the definition on the lengths of the intervals in Q′, the tasks corresponding to those intervals would have length of at most *w* and could fit into one batch. Since there are *b* empty intervals, this implies a partitioning of the tasks into *b* batches, which satisfies the scheduling problem.

Let $\left(p_{0},p_{1},\ldots ,p_{m}\right)$ be the solution of the *P*-value problem under $H_{0}^{GS}$ for *Q*. By definition, $p_{0}-p_{1}$ is the probability under $H_{0}^{GS}$ of generating a set of intervals $Q_{\text{rand}}$ with $K(R,Q_{\text{rand}})=0$. We observe that $p_{0}>p_{1}$ iff there exists at least one interval set $Q_{\text{rand}}$ with $K(R,Q_{\text{rand}})=0$. Combining with our reduction, $p_{0}>p_{1}$ iff the scheduling problem has a solution. To wrap up, we have given a reduction, computable in polynomial time, that shows that if there is a polynomial time algorithm for the *P*-value under $H_{0}^{GS}$, then there is a polynomial time algorithm for the scheduling problem. Since the scheduling problem is NP-complete, this implies that the *P*-value problem under $H_{0}^{GS}$ is NP-hard.

## 4 Sampling algorithm under $H_{0}^{GS}$

In this section we present Algorithm 1 which samples annotations from the gold standard null distribution ($H_{0}^{GS}$) in O(|Q| log L) time per sample. A fast-sampling algorithm serves at least two purposes. First, when the *P*-value is not too small, the algorithm can approximate it with a reasonable number of samples. Second, even if the *P*-value is small, the algorithm can provide the ground truth for comparing the fidelity of an alternate null hypothesis to $H_{0}^{GS}$.

If the weight of the query is low, rejection sampling (Devroye, 1986) can be used. This scheme places the start of each interval uniformly at random; if the resulting set of intervals is non-intersecting and separated, the sample is kept; if not, it is rejected. However, when the weight of the query is high, rejection dominates and the number of attempts needed is prohibitively high. We therefore pursue a rejection-free approach.

**Algorithm 1** Sample from $H_{0}^{GS}$  *Input*: Genome length *L* and an annotation *Q Output*: An annotation $Q_{\text{rand}}$, drawn from $H_{0}^{GS}$  *Notation*: BB(t,α,β) is the Beta-Binomial distribution with parameters *t* (i.e. the number of trials), *α* and *β*. It gives an integer between 0 and *t*. 1: O[1…|Q|]← a permutation of *lens*(*Q*) chosen uniformly at random

2: Gaps[0…|Q|]←(0,1,…,1,0)

3: freeSpaceRemaining←L−weight(Q)−|Q|+1

4: **for** *i* = 0 to |Q|−1  **do**

5:  x← sample from BB(freeSpaceRemaining,1,|Q|−i)

6:  Gaps[i]←Gaps[i]+x

7:  freeSpaceRemaining←freeSpaceRemaining−x

8: **end for**

9: Gaps[|Q|]←freeSpaceRemaining

10: $Q_{\text{rand}}\leftarrow$ annotation defined by placing intervals with lengths given by *O* onto the genome, with gaps between them defined by *Gaps*.

11: **return**  $Q_{\text{rand}}$

Algorithm 1 defines $Q_{\text{rand}}$ (Line 10) by first choosing the order *O* in which the required interval lengths appear (Line 1) and then setting the lengths of the |Q|+1 gaps (i.e. Gaps[0…|Q|]) such that their sum is L−weight(Q) (Lines 2–9). To force $Q_{\text{rand}}$ to be separated, it initializes each gap to be 1, except the first and last gap (Line 2). That leaves L−weight(Q)−|Q|+1 of *free space* to distribute among the |Q|+1 gaps (Line 3). It does this by sequentially processing all the gaps and, for each gap, sampling an integer *x* between 0 and the amount of remaining free space, increasing the gap by *x*, and decreasing the remaining free space by *x* (lines 5–7). To show that every $Q_{\text{rand}}$ is chosen with equal probability and in the necessary time, we will prove the following theorem:

Theorem 2. *Algorithm 1 outputs a sample from* $H_{0}^{GS}$  *in time* O(|Q| log L).

Before proving Theorem 2, we motivate the need for using the Beta-Binomial distribution to distribute the free space by considering two alternative ideas that are simpler but do not work. First, we could choose the free space for each gap by sampling uniformly from the amount of remaining free space. To see that this approach leads to unequal probabilities for different annotation, consider the example of lens(Q)={1,1} and *L* = 10. The probability of sampling the annotation {[3,4),[9,10)} is $\frac{1}{8\cdot 5}$, whereas the probability of sampling the annotation {[7,8),[9,10)} is $\frac{1}{8\cdot 1}$ Second, we could distribute the free space by taking a sample from the multinomial distribution with a number of trials equal to the free space, |Q|+1 categories, and category probabilities equal to $\frac{1}{|Q|+1}$. This approach also leads to non-uniform sampling of annotations; i.e. for the above example, the probability of sampling the two annotations is $\frac{7!}{3!4!0!}\cdot {\left(\frac{1}{3}\right)}^{7}\approx 0.016$ and $\frac{7!}{7!0!0!}\cdot {\left(\frac{1}{3}\right)}^{7}\approx 0.0004$, respectively.

Proof of Theorem 2. We have already argued that Algorithm 1 outputs an annotation. To prove the theorem, we first prove that the output $Q_{\text{rand}}$ is chosen uniformly at random from all annotations with $\mathrm{lens}(Q_{\text{rand}})=\mathrm{lens}(Q)$ and, second, that Algorithm 1 runs in time O(|Q| log L).

Distributing the free space among the |Q|+1 gaps can be restated as finding a *b*-partition. *A b-partition* of a non-negative integer *U* is an ordered *b*-tuple $\left(u_{1},\ldots ,u_{b}\right)$ of non-negative integers whose sum is equal to *U*. In our case, b=|Q|+1 and *U* is the free space. Note that for every permutation of the interval lengths, the number of annotations $Q_{\text{rand}}$ such that $\mathrm{lens}(Q_{\text{rand}})=\mathrm{lens}(Q)$ is identical and equal to the number of (|Q|+1)-partitions of the free space. Therefore, to sample $Q_{\text{rand}}$ uniformly, it suffices to first sample the permutation uniformly and then sample the *b*-partition uniformly. It remains to show that Algorithm 1 chooses a *b*-partition uniformly at random.

To count the total number of *b*-partitions of *U*, we can think of inserting *b* – 1 separators into a number line of length *U*, resulting in a sequence of U+b−1 slots with breakers assigned to *b* – 1 of those slots. The number of partitions is thus the number of ways to choose *b* – 1 slots out of U+b−1, which we denote as $S(U,b):=\left(\begin{array}{c} U+b-1\\ b-1 \end{array}\right)$.

It is useful to redefine a *b*-partition of *U* recursively as follows. For *b* = 1, $\left(u_{1}\right)$ is a *b*-partition of *U* iff $u_{1}=U$. For *b* > 1, $\left(u_{1},\ldots ,u_{b}\right)$ is a *b*-partition of *U* iff $u_{1}\in \{0,\ldots ,U\}$ and $\left(u_{2},\ldots ,u_{b}\right)$ is a (b−1)-partition of $U-u_{1}$. Observe that the number of *b*-partitions with $u_{1}=i$ is S(U−i,b−1). Therefore, to sample a *b*-partition uniformly in this recursive manner, we need to choose a value of *u*_1_ such that the chosen value is *i* with probability $\frac{S(U-i,b-1)}{S(U,b)}$. We can equivalently rewrite this (the algebraic derivation is in the Supplementary Section S1) as sampling *u*_1_ from the following cumulative distribution function:

(1) $$\text{Pr}[u_{1}\leq i]=1-\frac{(U-i+b-2)!U!}{(U-i-1)!(U+b-1)!}.$$

This distribution on *u*_1_ is the Beta-Binomial distribution with parameters *α* = 1, β=b−1, and *U* trials. Sampling from this distribution is indeed what the algorithm does (Line 5), thus the algorithm samples a |Q|+1-partition of free space uniformly at random.

To prove the runtime of the algorithm, first observe that uniformly sampling a permutation of |Q| elements (Line 1), initializing *Gaps* (Line 2), and constructing $Q_{\text{rand}}$ (Line 10) can be done trivially in O(|Q|) time. All other operations besides sampling from the Beta-Binomial distribution (Line 5) can be done in constant time. Thus, the total runtime is O(|Q|) multiplied by the time for sampling from the Beta-Binomial distribution. This can be done using the technique of inverse transform sampling, using a binary search to compute the inverse transform [as described in Devroye (1986)]. Briefly, the technique works by first choosing a real number *y* uniformly between 0 and 1 and then finding the smallest *i* such that $y\leq \text{Pr}[u_{1}\leq i]$. The search for *i* can be done in O(log U) time using binary search, because the cumulative distribution function is monotone and can be evaluated in constant time using Equation (1). Thus, the total runtime of the algorithm is O(|Q| log U)=O(|Q| log L).

## 5 The Markov chain null hypothesis ($H_{0}^{MC}$)

Given the challenges of computing exact *P*-values under $H_{0}^{GS}$ (Sarmashghi and Bafna, 2019) and the fact that doing so is NP-hard in the general case (Theorem 1), we turn to a more tractable yet still faithful null hypothesis. We are inspired by the somewhat parallel problem of generating random strings that have desired nucleotide frequencies (Robin *et al.*, 2005). In what is called the permutation model, the nucleotide frequencies in the generated strings must be exactly the desired ones, while in the more relaxed Markov chain model, the nucleotide frequencies in the generated strings must be the desired ones only in expectation. Many quantities turn out to be much simpler to compute in the Markov chain model than in the permutation model, making the relaxation a good tradeoff in many instances (Robin *et al.*, 2005). We will show that the same idea carries over to our problem as well.

An annotation can be generated using a two-state Markov chain as follows. The two states 0 and 1 correspond to being outside of an interval and inside an interval, respectively. The transition probabilities are given by $T=\left(\begin{array}{cc} t_{00} & t_{10}\\ t_{01} & t_{11} \end{array}\right)$, where, where *t_ij_* is the probability of transitioning from state *i* to state *j*. Let $\vec{\pi }=\pi_{0},\ldots ,\pi_{L-1}$ be the states of this Markov chain, after *L* steps. For simplicity, the initial state *π*_0_ and the last state $\pi_{L-1}$ are assumed to be 0. This sequence of states induces an interval set by combining all contiguous stretches of 1 states. For example, a sequence of states (0,0,0,1,1,1,0,1,1,0,1,0) would produce an interval set {[3,6),[7,9),[10,11)}.

The *Markov chain null hypothesis (*$H_{0}^{MC}$) is that the query is generated by this Markov chain with parameters

$$T=\left(\begin{array}{cc} 1-t_{01} & \frac{\left|Q\right|}{L-\mathrm{weight}(Q)-1}\\ \frac{\left|Q\right|}{\mathrm{weight}(Q)} & 1-t_{10} \end{array}\right).$$

Using a standard frequency counting approach (Koller and Friedman, 2009), these are the weights that maximize the probability of observing the given query set *Q*. Moreover, the expected length of the intervals and the gaps under $H_{0}^{MC}$ is asymptotically (with growing *L* and *n*) equal to the average lengths of the intervals and the gaps in *Q*, respectively (Koller and Friedman, 2009).

## 6 Algorithm for the *P*-value problem under $H_{0}^{MC}$

In this section, we give an algorithm (which we call MCDP) for the *P*-value problem under $H_{0}^{MC}$. In particular, given a genome length *L*, a reference annotation *R*, and a query annotation *Q*, the problem is to output $\left(p_{0},\ldots ,p_{m}\right)$, where:

$$p_{k}={\text{Pr}}_{Q_{\text{rand}}∼H_{0}^{MC}}[K(R,Q_{\text{rand}})\geq k].$$

We first need some definitions. Recall that n=|Q| and m=|R|. Let $R=\{[b_{1},e_{1}),\ldots ,[b_{m},e_{m})\}$ be the reference interval set. Let us also define $e_{0}:=1$ (note we set $e_{0}=1$ in order to accommodate for the fact *π*_0_ is always 0 in our definition) and $b_{m+1}:=L$. Let $g_{j}:=b_{j}-e_{j-1}$, for 1≤j≤m+1, and let $l_{j}:=e_{j}-b_{j}$, for 1≤j≤m. These are the lengths of the gaps and the intervals in *R*, respectively. We define $Z_{\text{any}}(i\overset{t}{\to }i\prime )$ as the probability of being in state i′ given that *t* positions earlier the state was *i*. We define $Z_{\text{zeros}}(i\overset{t}{\to }i\prime )$ as the probability that the next *t* states are all zero, with the last of these states being i′, conditioned on the current state being *i*. Naturally, if i′=1 and t≥1, then this probability is 0. We postpone the computation of $Z_{\text{any}}$ and $Z_{\text{zeros}}$ until Section 6.2.

### 6.1 Dynamic programming: simple form

Our algorithm for computing the *P*-value under $H_{0}^{MC}$ is based on dynamic programming, building upon some of the ideas of the algorithm of Sarmashghi and Bafna (2019). It builds a table of entries $P_{\text{DP}}[j,\kappa ,s]$, for 0≤j≤m, 0≤κ≤m and s∈{0,1}. $P_{\text{DP}}[j,\kappa ,s]$ denotes the probability of hitting (i.e. intersecting) exactly *κ* reference intervals after generating *e_j_* states of the Markov chain (i.e. finishing at the last base of *j*th reference interval, which is $e_{j}-1$), with the last generated state being *s*. From this table, we can compute *p_k_* as follows:

$$\begin{array}{c} \text{Pr}[K(R,Q_{\text{rand}})\geq k]=\sum_{\kappa =k}^{m}\text{Pr}[K(R,Q_{\text{rand}})=\kappa ]\\ =\sum_{\kappa =k}^{m}P_{\text{DP}}[m,\kappa ,0]+P_{\text{DP}}[m,\kappa ,1]. \end{array}$$

The base cases of $P_{\text{DP}}$ follow from definitions: $P_{\text{DP}}[j,\kappa ,s]=1$ when j=κ=s=0 and $P_{\text{DP}}[j,\kappa ,s]=0$ when either 1) j=κ=0 and *s* = 1, 2) j<κ, or 3) κ<0. The general case recurrence, which we explain below, is given by:

(2) $$\begin{array}{c} P_{\text{DP}}[j,\kappa ,s]=P_{\text{DP}}[j-1,\kappa ,0]P_{\text{nohit}}(j,0,s)\\ \;\;+P_{\text{DP}}[j-1,\kappa ,1]P_{\text{nohit}}(j,1,s)\\ \;\;+P_{\text{DP}}[j-1,\kappa -1,0]P_{\text{hit}}(j,0,s)\\ \;\;+P_{\text{DP}}[j-1,\kappa -1,1]P_{\text{hit}}(j,1,s). \end{array}$$

To understand this recurrence, we need to define the functions $P_{\text{nohit}}$ and $P_{\text{hit}}$. We define $P_{\text{nohit}}(j,i,i\prime )$ as the probability that $\vec{\pi }$ does not hit the *j*th reference interval and is in state i′ at position $e_{j}-1$, given that the state at position $e_{j-1}-1$ is *i*. In other words, it is the probability that $\pi_{x}=0$ for $b_{j}\leq x<e_{j}$ and $\pi_{e_{j}-1}=i\prime$, given that $\pi_{e_{j-1}-1}=i$. This can happen in one of two ways: either $\pi_{b_{j}-1}$ is zero or one. In either case, we need to take *g_j_* steps to transition from *i* to $\pi_{b_{j}-1}$, using any intermediate states, and then *l_j_* steps to transition from $\pi_{b_{j}-1}$ to i′, using only zero states. Thus,

$$P_{\text{nohit}}(j,i,i\prime )=Z_{\text{any}}(i\overset{g_{j}}{\to }0)Z_{\text{zeros}}(0\overset{l_{j}}{\to }i\prime )+Z_{\text{any}}(i\overset{g_{j}}{\to }1)Z_{\text{zeros}}(1\overset{l_{j}}{\to }i\prime ).$$

In a parallel fashion, we define $P_{\text{hit}}(j,i,i\prime )$ as the probability that $\vec{\pi }$ intersects the *j*th reference interval and is in state i′ at position $e_{j}-1$, given that the state at position $e_{j-1}-1$ is *i*. In other words, it is the probability that $\pi_{e_{j}-1}=i\prime$ and there exists $x\in \{b_{j},\ldots ,e_{j}-1\}$ such that $\pi_{x}=1$, given that $\pi_{e_{j-1}-1}=i$. This can be calculated as the probability of going from *i* to i′ using any states minus the probability of going from *i* to i′ in a way that does not hit the *j*th interval. Thus,

$$P_{\text{hit}}(j,i,i\prime )=Z_{\text{any}}(i\overset{g_{j}+l_{j}}{\to }i\prime )-P_{\text{nohit}}(j,i,i\prime )$$

Now we can justify the general dynamic programming recurrence (Equation 2). There are two ways that we can hit exactly *κ* of the first *j* reference intervals. Either, we have hit *κ* of the first *j* – 1 intervals and then do not hit the *j*th interval, or we have hit κ−1 of the first *j* – 1 intervals and then hit the *j*th interval.

### 6.2 Dynamic programming: efficient formulation

Computing the simple dynamic programming table given by Equation (2) reduces to repeatedly evaluating $Z_{\text{any}}$ and $Z_{\text{zeros}}$. In this subsection, we show how to efficiently do this by reformulating the dynamic programming table using matrix notation.

The probability given by $Z_{\text{any}}(i\overset{a}{\to }i\prime )$ can be written compactly by taking the value in row *i* and column *j* of the matrix obtained by raising *T* to the *a*th power (Norris, 1998). In other words, $Z_{\text{any}}(i\overset{a}{\to }i\prime )={\left(T^{a}\right)}_{ii\prime }.$ For $Z_{\text{zeros}}(i\overset{a}{\to }i\prime )$, we must modify the transition matrix to forbid any transitions into the 1 state by setting those probabilities to zero. That is, we define $T_{\text{mod}}:=\left(\begin{array}{cc} t_{00} & 0\\ t_{10} & 0 \end{array}\right)$ and $Z_{\text{zeros}}(i\overset{a}{\to }i\prime )={\left(T_{\text{mod}}{}^{a}\right)}_{ii\prime }.$

Using these definitions of *Z*, we can more naturally express $P_{\text{nohit}}$ using a dot product, i.e. $P_{\text{nohit}}(j,i,i\prime )={\left(T^{g_{j}}\cdot T_{\text{mod}}{}^{l_{j}}\right)}_{ii\prime }$. Similarly, we can write $P_{\text{hit}}(j,i,i\prime )={\left(T^{g_{j}+l_{j}}-T^{g_{j}}\cdot T_{\text{mod}}{}^{l_{j}}\right)}_{ii\prime }={\left(T^{g_{j}}\cdot (T^{l_{j}}-T_{\text{mod}}{}^{l_{j}})\right)}_{ii\prime }.$

Given that we can express $P_{\text{nohit}}$ and $P_{\text{hit}}$ using matrix operations, it becomes more natural to view the dynamic programming table $P_{\text{DP}}$ as a 2D table $P_{\text{DP}2}[j,\kappa ]$ with vectors of length 2 as its cells. The base cases of this table are given by: $P_{\text{DP}2}[j,\kappa ]=\left(\begin{array}{cc} 1 & 0 \end{array}\right)$ when j=κ=0, and $P_{\text{DP}2}[j,\kappa ]=\left(\begin{array}{cc} 0 & 0 \end{array}\right)$ when j<κ or κ<0. The general case is now given by:

$$\begin{array}{c} P_{\text{DP}2}[j,\kappa ]=P_{\text{DP}2}[j-1,\kappa ]\cdot T^{g_{j}}\cdot T_{\text{mod}}{}^{l_{j}}\\ \;\;+P_{\text{DP}2}[j-1,\kappa -1]\cdot T^{g_{j}}\cdot (T^{l_{j}}-T_{\text{mod}}{}^{l_{j}}). \end{array}$$

To efficiently compute the required matrix exponentiation, we observe that *T* and $T_{\text{mod}}$ are both diagonizable. This allows them to be exponentiated in just two matrix multiplications (Margalit and Rabinoff, 2017):

$$\begin{array}{c} T^{a}=\left(\begin{array}{cc} 1 & \frac{-t_{01}}{t_{10}}\\ 1 & 1 \end{array}\right)\left(\begin{array}{cc} 1 & 0\\ 0 & {\left(t_{00}-t_{10}\right)}^{a} \end{array}\right)\left(\begin{array}{cc} \frac{t_{10}}{t_{01}+t_{10}} & \frac{t_{01}}{t_{01}+t_{10}}\\ \frac{-t_{10}}{t_{01}+t_{10}} & \frac{t_{10}}{t_{01}+t_{10}} \end{array}\right)\\ T_{\text{mod}}{}^{a}=\left(\begin{array}{cc} t_{00}{}^{a} & 0\\ -t_{10}t_{00}{}^{a-1} & 0 \end{array}\right) \end{array}$$

This calculation takes constant time, since our matrices have constant size (i.e. 2 rows by 2 columns). To reduce computational overhead when the exponent is small (a≤50), we use the simpler exponentiation by squaring technique (Gordon, 1998).

Finally, we have that the time complexity of the dynamic programming is $O(m^{2})$, since each cell takes O(1) time to compute. Combined with the O(n) time to compute the parameters of the Markov chain, the total time complexity of our algorithm for computing *P*-values under $H_{0}^{MC}$ is $O(m^{2}+n)$. The memory complexity is O(m), since only the last two rows and the last column are needed to be held in memory.

## 7 Experimental results

We implemented MCDP in Python and made it available open-source at https://github.com/fmfi-compbio/mc-overlaps. All the datasets used for evaluation are also available at https://github.com/fmfi-compbio/mc-overlaps-reproducibility.

### 7.1 Evaluated algorithms and metrics

In order to examine the performance of MCDP, we compare SBDP, the order-restricted dynamic programming algorithm of Sarmashghi and Bafna (2019). We do not compare to the Poisson Binomial Approximation algorithm of Sarmashghi and Bafna (2019), since they show it to be substantially less accurate in practice. We also do not compare with alternate approaches that ignore interval lengths, since they were shown to be inaccurate in many cases (Sarmashghi and Bafna, 2019). To evaluate accuracy with respect to $H_{0}^{GS}$, we use Algorithm 1 with 10 000 samples. We conducted experiments on a server with Intel^®^ Xeon^®^ CPU E5-2680 v3 at 2.50 GHz × 48 and 96 GB DDR4 RAM, using a single thread.

### 7.2 Datasets

*Synthetic data:* To simulate a chromosome annotation, we have parameters specifying the chromosome length, the number of intervals and the desired coverage. *Coverage* is the ratio of the sum of the interval lengths to the chromosome length. All interval lengths are identical and set to the coverage times the chromosome length divided by the number of intervals. The locations of the intervals are chosen uniformly at random using Algorithm 1. We chose to have fixed-length intervals in order to limit the dimension of our evaluation space; however, we note that this choice does not favor MCDP, since $H_{0}^{MC}$ actually gives a geometric distribution of interval lengths.

*Real datasets from Sarmashghi and Bafna (2019):*  Sarmashghi and Bafna (2019) evaluated SBDP on four real datasets which cover diverse biological applications, summarized in Table 1. All datasets use the multi-chromosome hg19 human genome assembly. In order to run MCDP, we merged any intersecting or non-separate intervals. Note that we combine the *P*-value solution for individual chromosomes into a *P*-value solution for multiple-chromosomes using the post-processing algorithm described in Sarmashghi and Bafna (2019); it runs in time $O(Nm^{2})$ and memory O(Nm), where *N* is the number of chromosomes.

**Table 1.** Description of real datasets

Dataset	Reference (*R*)					Query (*Q*)					*K*(*R*, *Q*)
	n. unmerged	n. intervals	Average interval size	Average gap size	Coverage	n. unmerged	n. intervals	Average interval size	Average gap size	Coverage	
*EC*	6521	101	2 408 032	27 965 355	0.079	247	116	4 186 642	22 307 922	0.157	54
*CS*	4994	4994	611	619 144	0.001	59 758	27 180	4241	109 650	0.037	344
*CNV*	13 948	9342	29 060	302 280	0.088	3132	3132	35 582	952 517	0.036	1009
*H3K4me3*	59 758	24 888	8792	115 587	0.071	19 538	19 538	1940	158 176	0.012	2642

*Notes*: Column ‘n. unmerged’ denotes the number of intervals in the original input file, before we merged non-separated intervals.

The first dataset (labeled *EC*) uses copy number amplifications that are recurrent in The Cancer Genome Atlas as the reference annotation and regions enriched for extra-chromosomal DNA sequence as the query annotation (Turner *et al.*, 2017). In the second dataset (labeled *CS*), the reference annotation is the set of regions that were assigned a certain chromatin state (9) by a computational study of Ernst and Kellis (2010) and the query annotation is the set of promoter regions obtained from RefSeq (Sarmashghi and Bafna, 2019). The third dataset (labeled *CNV)* has the set of all non-coding genes as the reference annotation and the set of all regions with copy number gains as the query annotation (Zarrei *et al.*, 2015). The fourth dataset (labeled *H3K4me3*) has the set of promoter regions as the reference annotation and regions highly enriched for H3K4me3 histone modification as the query annotation (Guenther *et al.*, 2007).

Sarmashghi and Bafna (2019) showed that the SBDP *P*-values in these datasets are significant, indicating an association between two biological mechanisms and suggesting future directions of study. Although we will show that MCDP gives different *P*-values, they will still be significant. We focus our analysis and discussion on the runtime, memory usage and accuracy of our tools, rather than the biological interpretation of the significance that was already discussed by Sarmashghi and Bafna (2019).

*Real datasets from Zarrei et al. (2015* *; Fig. 3b):* This study looked at the significance of the overlap between copy number losses in the human genome (23 438 intervals) and all exons from RefSeq-annotated human genes (217 527 intervals; Zarrei *et al.*, 2015; Fig. 3b). They further grouped the genes according to properties of interest such as being non-coding or being involved in cancer. Supplementary Table S1 shows the properties of these annotations, and Supplementary Table S4 gives a description of the biological significance of each gene group. We use the copy number loss annotation as the reference and the various gene groups as the query. Unfortunately, a small fraction of the gene names used in the original study could not be located in RefSeq and, for the rest, the gene coordinates in the current RefSeq are possibly different from the time of the original study; therefore, our annotations are not identical.

### 7.3 MCDP is more efficient than SBDP

*Synthetic data:*  Table 2 shows the runtime and memory comparison of MCDP and SBDP on synthetic data. We have varied the size of chromosome as well as the number of intervals in the reference and query, since these are the parameters that appear in the theoretical time complexity of these algorithms. The empirical runtime of MCDP is consistent with the theoretical predictions of $O(m^{2}+n)$, i.e. it is essentially independent of the chromosome length *L* and the dependence on the size of the query (*n*) is dominated by the dependence on the size of the reference (*m*). The memory usage, on the other hand, seems empirically to be fairly constant, even though the theory predicts it to be O(m). On further investigation, we found that running MCDP on an empty input takes around 130 MB, implying that the heap memory used by MCDP is negligible compared with the baseline Python overhead.

**Table 2.** Running time and peak memory consumption of MCDP and SBDP on synthetic data

Simulation parameters			Time (min)		Memory (MB)	
L	|R|	|Q|	MCDP	SBDP	MCDP	SBDP
10^5^	50	50	<1	<1	137	84
10^5^	50	5000	<1	17	139	93
10^5^	5000	50	5	11	139	4039
10^5^	5000	5000	5	—	142	—
10^6^	50	50	<1	2	136	453
10^6^	50	5000	<1	168	138	455
10^6^	5000	50	8	114	139	39 908
10^6^	5000	5000	9	—	140	—
10^7^	50	50	<1	16	137	4140
10^7^	50	5000	<1	—	138	—
10^7^	5000	50	8	—	139	—
10^7^	5000	5000	8	—	141	—
10^8^	50	50	<1	156	137	41 009
10^8^	50	5000	<1	—	139	—
10^8^	5000	50	9	—	139	—
10^8^	5000	5000	10	—	141	—

*Notes*: The coverage of both the reference and the query is fixed to 0.2. No scaling was used for SBDP. Dashes (—) indicate that the experiment either ran for more than 4 h or exceeded the memory capacity of our machine (∼100 GB).

In general, the results show that MCDP can be comfortably used with chromosomes of any length and with all the tested interval annotations. On the other hand, SBDP (without scaling) uses orders of magnitude more running time and memory which quickly becomes prohibitive with an increase in any of the parameters. For example, the analysis of a human-length chromosome (i.e. 10^8^ bp) with only 50 reference and query intervals already takes ∼41 GB of memory and more than 2 h, while MCDP takes <1 min and less than half a GB of RAM. When the number of intervals in either the reference or query annotation is increased, SBDP exceeds the memory capacity of our machine (∼100 GB); moreover, the extrapolated runtime (based on the theoretical scaling of O(nmL)) is more than 10 days. Note that these results do not invalidate the SBDP approach but rather motivate that a scaling factor usually needs to be applied before running SBDP.

*Real datasets from Sarmashghi and Bafna (2019):*  Table 3 shows the run time and memory usage on the real datasets from Sarmashghi and Bafna (2019). In order to run SBDP, we needed to use a scaling factor no larger than $\nu =10^{-3}$; we also tried $\nu =10^{-4}$, which has a runtime which may be deemed more practical for some of the datasets. We did not use a smaller scaling factor since, as we will show in Section 7.4, the accuracy of SBDP already begins to deteriorate with $\nu =10^{-4}$.

**Table 3.** Running time and peak memory usage on the four real datasets from Sarmashghi and Bafna (2019)

Dataset	Time (min)			Memory (MB)		
	MCDP	SBDP $\nu =10^{-4}$	SBDP $\nu =10^{-3}$	MCDP	SBDP $\nu =10^{-4}$	SBDP $\nu =10^{-3}$
			
*EC*	<1	<1	<1	<1	60	214
*CS*	<1	51	534	<1	209	1204
*CNV*	2	9	85	<1	963	9153
*H3K4me3*	11	152	1554	<1	2563	2650

The running time of MCDP is roughly one order of magnitude less than SBDP with $\nu =10^{-4}$, and two orders of magnitude less than SBDP with $\nu =10^{-3}$. For the largest dataset (*H3K4me3*), MCDP took 11 min, whereas SBDP took 25 (respectively, 2.5) h with $\nu =10^{-3}$ (respectively, $10^{-4}$). We conclude that on large datasets, MCDP is faster and more memory efficient than SBDP even after scaling.

### 7.4 MCDP is more accurate than SBDP with practical scaling

*Synthetic data:* We generated synthetic data by varying the number of intervals in the reference and query and the length of the query intervals; we kept the genome length and the reference interval length constant. Table 4 shows the predicted critical value (i.e. the value of *K*(*R*, *Q*) for which *P* = 0.05) for MCDP and SBDP with various scaling parameters. For completeness, we also include measurements of Kullback–Leibler divergence and mean square bias (Supplementary Table S2) in the Supplementary Appendix, though the numbers are harder to interpret. To better make summary observations, Table 4 highlights the simulation parameters under which the critical value is at least 10% different from the sampled $H_{0}^{GS}$. We see that MCDP is accurate in all but two of the cases (Lines 25 and 26 in Table 4). These cases belong to the only group (|R|=2500 and $\ell_{q}=1000)$ where a query interval is expected to cover more than one reference interval. This group pushes to the limit the assumption of our underlying Markov chain model, that the query interval lengths are geometrically distributed. As a result, MCDP’s PMF is more heavy-tailed than the sampled one (Supplementary Fig. S1).

**Table 4.** Critical value on significance level 0.05 on synthetic data

Simulation parameters					Critical value				
Ref		Query			$H_{0}^{GS}$	MCDP	SBDP(*ν*)		
|R|	Coverage	*l_q_*	|Q|	Coverage			$10^{-3}$	$10^{-2}$	$10^{-1}$
50	0.005	10	500	0.005	6	6	*26*	6	6
50	0.005	10	5000	0.05	29	29	*51*	27	27
50	0.005	10	50 000	0.5	51	51	51	51	—
50	0.005	100	50	0.005	3	3	*6*	2	3
50	0.005	100	500	0.05	10	10	*27*	*6*	9
50	0.005	100	5000	0.5	46	46	51	*32*	45
50	0.005	1000	5	0.005	2	2	2	2	2
50	0.005	1000	50	0.05	7	7	6	6	7
50	0.005	1000	500	0.5	34	34	32	32	34
500	0.05	10	500	0.005	36	36	*213*	33	33
500	0.05	10	5000	0.05	237	238	*501*	223	—
500	0.05	10	50 000	0.5	501	501	501	—	—
500	0.05	100	50	0.005	10	10	*27*	*6*	9
500	0.05	100	500	0.05	61	62	*219*	*34*	58
500	0.05	100	5000	0.5	422	423	*501*	*269*	—
500	0.05	1000	5	0.005	7	8	*5*	6	7
500	0.05	1000	50	0.05	37	40	*27*	34	36
500	0.05	1000	500	0.5	292	296	*220*	269	290
2500	0.25	10	500	0.005	150	153	*489*	139	—
2500	0.25	10	5000	0.05	1129	1133	*2492*	—	—
2500	0.25	10	50 000	0.5	2501	2501	—	—	—
2500	0.25	100	50	0.005	32	35	*51*	*19*	31
2500	0.25	100	500	0.05	267	274	*489*	*142*	—
2500	0.25	100	5000	0.5	2067	2072	*2501*	—	—
2500	0.25	1000	5	0.005	19	*31*	*6*	20	19
2500	0.25	1000	50	0.05	153	*184*	*51*	144	152
2500	0.25	1000	500	0.5	1401	1440	*489*	1290	—

*Notes*: SBDP is run with three different scaling values, $\nu =10^{-1},10^{-2},10^{-3}$. The chromosome length is fixed to $L=10^{6}$ in order to be able to evaluate SBDP accuracy for scaling values as mild as $\nu =10^{-1}$. The lengths of the reference intervals are fixed to 100 and the length of query intervals is *l_q_*. Each time value corresponds to the average critical value over 10 annotation replicates, where each replicate corresponds to a different random sample of annotations *R* and *Q* with the specified parameters. The standard error of the critical values is at most 9% of their mean, for all combinations of methods and simulation parameters. Average critical values that differ by more than 10% from the average $H_{0}^{GS}$ values are highlighted in italic blue.

When compared with SBDP, MCDP is substantially more accurate at $\nu =10^{-3}$ and even at $\nu =10^{-2}$. At $\nu =10^{-1}$, SBDP is more accurate than MCDP, indicating that the improved accuracy of MCDP over SBDP is due to its computational efficiency rather than a more accurate algorithm. Note however that running SBDP with $\nu =10^{-1}$ is infeasible for most human-scale datasets.

**Fig. 2.**
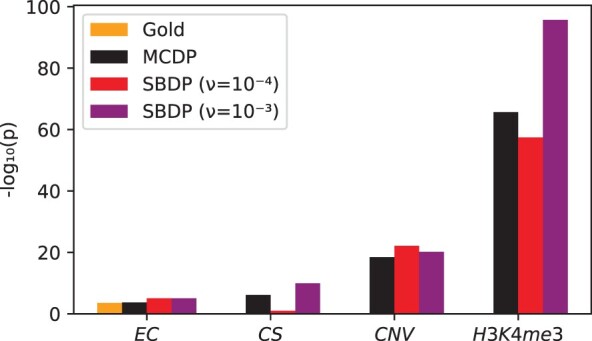
*P*-values for the real datasets from Sarmashghi and Bafna (2019), shown on a negative log scale. On all datasets except *EC*, the sampled $H_{0}^{GS}$ is not shown because every one of the 10 000 samples had less than *K*(*R*, *Q*) overlaps; the *rule of three* (Burns, 1983) implies that in this case, with 10 000 samples, the true *P*-value can only be said to be less than $P<3\times 10^{-4}$ with 95% probability

**Fig. 3.**
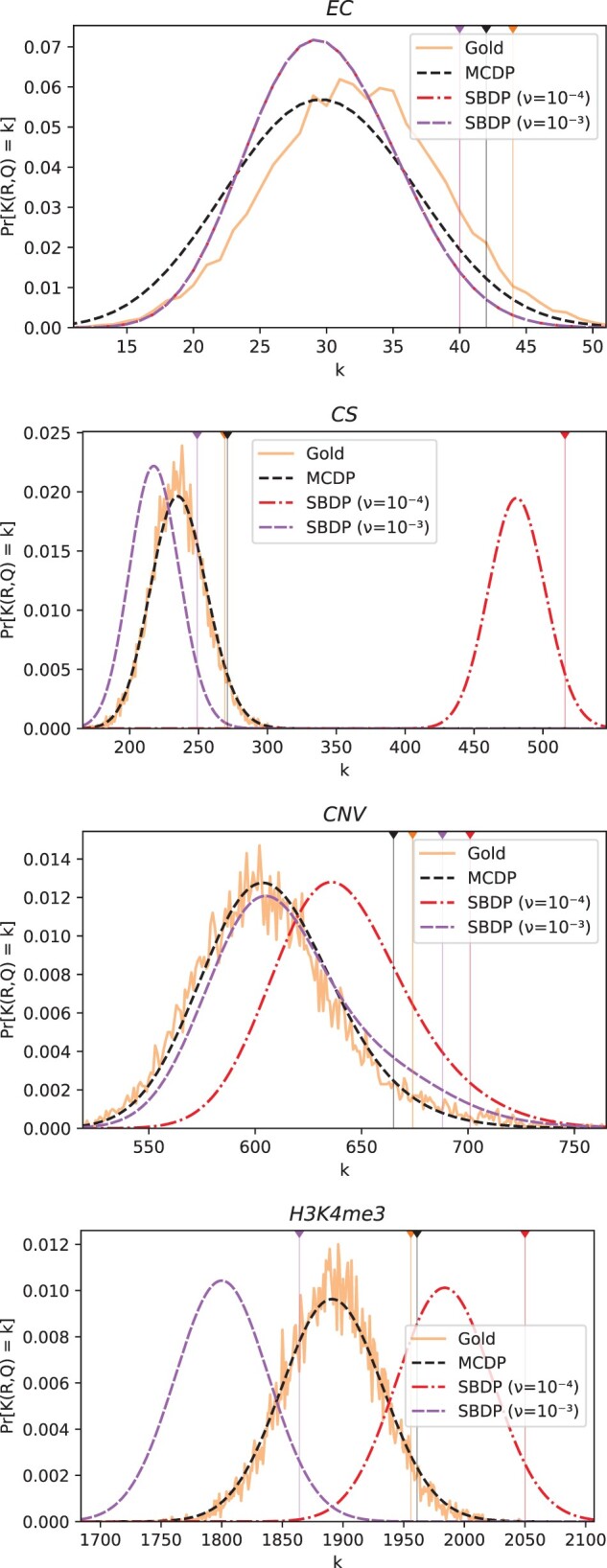
Probability mass functions for the overlap statistics in the four datasets from Sarmashghi and Bafna (2019). The vertical lines show the critical values for significance level 0.05. Note that the *x*-axis is zoomed in around the main probability mass. The critical value is the smallest value of *k* for which Pr[K(R,Q)≥k]≤0.05. Supplementary Table S3 contains the raw critical values

*Real datasets from Sarmashghi and Bafna (2019):*  Figure 2 shows the *P*-values computed by MCDP and SBDP, compared with sampled $H_{0}^{GS}$  (Sarmashghi and Bafna, 2019). We use $\nu =10^{-4}$ and $\nu =10^{-3}$ for SBDP, since larger scaling values exceeded our runtime cutoff (4 h) and/or server memory. For the *EC* dataset, we see that MCDP is closer to $H_{0}^{GS}$ than SBDP with either scaling factor. For the other three datasets, the *P*-values are too small to be computed by sampling. To better understand the accuracy in these cases, we plotted the probability mass function generated by all the approaches (Fig. 3). Overall, MCDP produces a distribution with a mode closer to the gold standard than does SBDP. In particular, for $\nu =10^{-3}$, while the modes of MCDP and of SBDP are both roughly accurate for *EC* and *CNV*, the modes of MCDP are better for *H3K4me3* and *CS*. For $\nu =10^{-4}$, SBDP’s modes are substantially shifted for all datasets except *EC*.

Figure 3 also shows the critical value for each dataset. MCDP is always closer to the true value, with an error of at most 5%. SBDP with scaling factor $\nu =10^{-3}$ is slightly worse but still close, whereas SBDP with scaling factor $\nu =10^{-4}$ shows major discrepancies. In particular, there is a 90% error for *CS* that results in the observed overlap (K(R,Q)=344) being mistakenly deemed insignificant at level 0.05.

### 7.5 Large-scale study replication with MCDP

To demonstrate how MCDP enables the analysis of large datasets, we replicated the enrichment/depletion analysis from Zarrei *et al.* (2015; Fig. 3b). On the whole set of RefSeq exons (217 527 reference intervals), MCDP completes in <17 h (Supplementary Table S1). Running SBDP with a scaling factor of $\nu =10^{-3}$ exceeded the memory of our server (∼100 GB). We did not attempt $\nu =10^{-4}$ since, as we saw in Section 7.4, this gives inaccurate *P*-values in nearly all our tests.

**Fig. 4.**
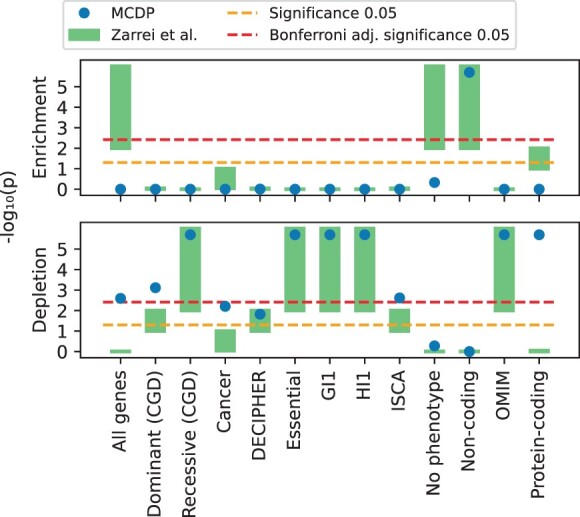
Replication of Zarrei *et al.* (2015; Fig. 3b) analysis, where we use copy number losses as a reference and each of the columns as a separate query. The columns correspond to various subset of exons from RefSeq human genes; the exact descriptions are in Supplementary Table S4. The two panels show *P*-values for enrichment and depletion of copy number losses in the corresponding gene column. The blue dots represent the *P*-values produced by MCDP, the green boxes represent the *P*-value range reported by Zarrei *et al.* (2015). We clip the *y*-axis at the top, and the points and ranges at those boundaries actually have more extreme values. The orange dashed lines (lower) correspond to significance level 0.05, and the red dashed lines (upper) correspond to Bonferroni-adjusted significance level (i.e. $\frac{0.05}{13}$, since there are 13 experiments in this case). Note that MCDP can report the *P*-value for both enrichment and depletion because it computes the probability mass function for all values of *K*(*R*, *Q*) (A color version of this figure appears in the online version of this article)

Zarrei *et al.* (2015) measured the significance of enrichment or depletion using sampling (though the details of the sampling strategy were unclear to us; Fig. 3b) and reported a range for the *P*-values. Figure 4 shows the *P*-values obtained by MCDP in comparison with the ranges given by Zarrei *et al.* (2015). The *P*-values are generally similar but in three cases MCDP suggests depletion and the original analysis enrichment. For the *Protein-coding* dataset this is caused by the changes in the underlying annotations, and for the *All genes* and *No phenotype* datasets the difference stems from the fact that Zarrei *et al.* use a different null hypothesis and measure the significance of the number of intersecting nucleotides whereas we look at the number of intersecting intervals. We discuss these cases further in Supplementary Section S2.

## 8 Conclusion

In this article, we have studied the problem of computing the significance (i.e. *P*-value) of the number of overlaps between two genome annotations. We have shown that the gold standard null hypothesis formulation ($H_{0}^{GS}$), implicitly assumed in other works (e.g. Ernst and Kellis, 2010; Zarrei *et al.*, 2015), leads to an NP-hard problem (Section 3). This motivated us to propose an alternative null hypothesis, based on Markov chains, which relaxes some of the constraints of $H_{0}^{GS}$ (Section 5). We have then devised an algorithm MCDP to compute *P*-values under this hypothesis in time $O(m^{2}+n)$ and space O(m) (Section 6). Unlike the previous approach of SBDP, which has O(νnmL) runtime and O(νmL) memory, MCDP’s resources are independent of the genome length, making it substantially more efficient on human annotations. To evaluate the accuracy of MCDP and SBDP against $H_{0}^{GS}$, we have derived an efficient direct sampling algorithm, which, to the best of our knowledge, is the first such algorithm that does not rely on rejection sampling (Section 4). Our experiments have shown that while SBDP can achieve higher accuracy than MCDP with respect to $H_{0}^{GS}$ by setting the scaling parameter *ν* closer to 1, doing so can result in prohibitive time and/or memory usage on human-scale datasets (Section 7). Overall, MCDP requires orders of magnitude less time and memory at comparable levels of accuracy.

We see various methodological directions to improve our algorithm. The first major direction is improving the run time of MCDP. Constant speedups can be obtained by porting the code from Python into C++ or by parallelizing it. Improving the quadratic dependence on the number of query intervals can potentially be done by employing the fast Fourier transform (Kozen, 1992), though it may negatively affect accuracy (Nagarajan *et al.*, 2005). The second direction is improving the accuracy of MCDP. Our $H_{0}^{MC}$ null hypothesis implicitly models the interval lengths by a geometric distribution, which may be suboptimal in some biological contexts. This can be solved within the MC framework using *discrete phase-type distributions*, which can fit a true distribution of interval lengths at the expense of running time (Isensee and Horton, 2005).

The Markov chain framework we have applied in this article lends itself to efficiently addressing the additional challenges that may arise in biological applications. For example, in the analysis of Zarrei *et al.* (2015), it might be more insightful to determine whether a specific group of genes has significantly more variants than an average gene, rather than a random subset of the whole genome. More generally, it might make sense to exclude certain regions (e.g. centromeres) from the annotations allowed by the null hypothesis (Kanduri *et al.*, 2019). One could also train different models for certain regions in order to account for a confounding factor (e.g. GC content) and chain these regions together. Another challenge is that in our current framework, we assign the same significance to an overlap of 1 nt as to an overlap of 1000 nt. In situations where this becomes a factor, one could either require a larger minimum overlap length (as done by Sarmashghi and Bafna (2019)) or use different colocalization metrics such as overlap coverage or Euclidean/cosine metrics (Gu *et al.*, 2021). Finally, some applications may require comparing annotations of a graphical pangenome rather than a single linear genome (Rand *et al.*, 2017). Some of these challenges have been addressed in various other frameworks, though not comprehensively and often *ad hoc* (Kanduri *et al.*, 2019). These challenges could potentially be naturally expressed in the language of Markov chains (by adjusting its structure) or other stochastic models (e.g. random walks), which could serve to unify the existing approaches. We see this bridge between annotation overlap significance and the deeply studied area of stochastic modeling as our main conceptual contribution.

## Supplementary Material

btac255_Supplementary_Data
