# Supplementary material for: Markov chains improve the significance computation of overlapping genome annotations
Source: Bioinformatics. 2022 Jun 27;38(Suppl 1):i203–11. doi: 10.1093/bioinformatics/btac255 (PMC9235476; doi:10.1093/bioinformatics/btac255)
Supplement: btac255_Supplementary_Data [file btac255_supplementary_data.pdf]

# Markov chains improve the significance computation of overlapping genome annotations
# *Supplementary material*

Askar Gafurov, Broňa Brejová and Paul Medvedev

## S1 Derivation of cumulative distribution function for sampling

In this section, we derive the cumulative distribution function of the first element in a $b$-partition, as described in the proof of Theorem 2. Our starting point is that $\Pr[u_1 = i] = \frac{S(U-i,b-1)}{S(U,b)}$, where $S(U,b) = \binom{U+b-1}{b-1}$. Then,

$$\Pr[u_1 \le i] = \frac{1}{S(U,b)} \sum_{j=0}^{i} S(U-j, b-1) = \frac{1}{S(U,b)} \sum_{j=0}^{i} \binom{U-j+b-2}{b-2} =$$
$$= 1 - \frac{1}{S(U,b)} \sum_{j=0}^{U-i-1} \binom{j+b-2}{b-2} = 1 - \frac{1}{S(U,b)} \sum_{j=b-1}^{U-i+b-2} \binom{j-1}{b-2}$$

We can apply the fact that for any positive integers $n$ and $k$, $\binom{n}{k} = \sum_{i=k}^{n} \binom{i-1}{k-1}$ (Graham *et al.*, 1994). Then,

$$\Pr[u_1 \le i] = 1 - \frac{1}{S(U,b)} \binom{U-i+b-2}{b-1} = 1 - \frac{(U-i+b-2)!U!(b-1)!}{(U-i-1)!(b-1)!(U+b-1)!} = 1 - \frac{(U-i+b-2)!U!}{(U-i-1)!(U+b-1)!}.$$

## S2 Comparison of the results in the dataset by Zarrei *et al.* (2015)

When we compare the results of the MCDP algorithm and the original analysis by Zarrei *et al.* (2015) shown in Figure 4, we see major discrepancies in three datasets: *Protein-coding*, *All genes* and *No phenotype*. In all three cases MCDP reports depletion (for *No phenotype* the depletion is not statistically significant), whereas Zarrei et al. report enrichment (for *Protein coding* the enrichment is only weakly significant).

Zarrei et al. base their analysis on the number of nucleotides in the overlap between the two annotations. They report that the proportion of bases in the whole genome covered by CNV loss regions is 7.5%, whereas within exons of protein-coding genes the proportion CNV loss bases is very slightly higher, leading to non-significant difference (Zarrei *et al.*, 2015, Fig 3b). The genome-wide coverage of losses was computed after exclusion of assembly gaps (e.g. centromeric regions), and without this exclusion the background proportion would be only 6.95%, suggesting even stronger enrichment of losses in exons of protein coding genes. As the exact set of exons from the study is not available, we use the exons from the present-day RefSeq genes whose gene names match those listed by Zarrei *et al.* (2015). Of these exons, only 6.73% is covered, which is depleted even compared to the 6.95% baseline. As MCDP also reports a significant depletion, we conclude that for the *Protein-coding* dataset the discrepancy from the original analysis is due to a different set of gene annotations.

This is not the case for the *All genes* and *No phenotype* datasets. Using our reconstructed annotations, the proportions of bases of exons covered in these datasets are 7.31% and 8.08%, respectively. This is more than the background proportion of 6.95%; Zarrei et al. also report enrichments compared to their background proportion. However, MCDP uses a different statistics based on the number of overlapping intervals rather than shared bases. For the *All genes* dataset the observed number of overlaps is 16,102 and the expected number of overlaps under $\mathcal{H}_0^{MC}$ is 17,072, which leads to a significant p-value for depletion. For the *No phenotype* dataset, the observed number of overlaps is 8,233, and the expected number is 8,218, which is a small and not statistically significant enrichment. In these cases we conclude that the discrepancy between the two methods stems mainly from the use of different statistics.

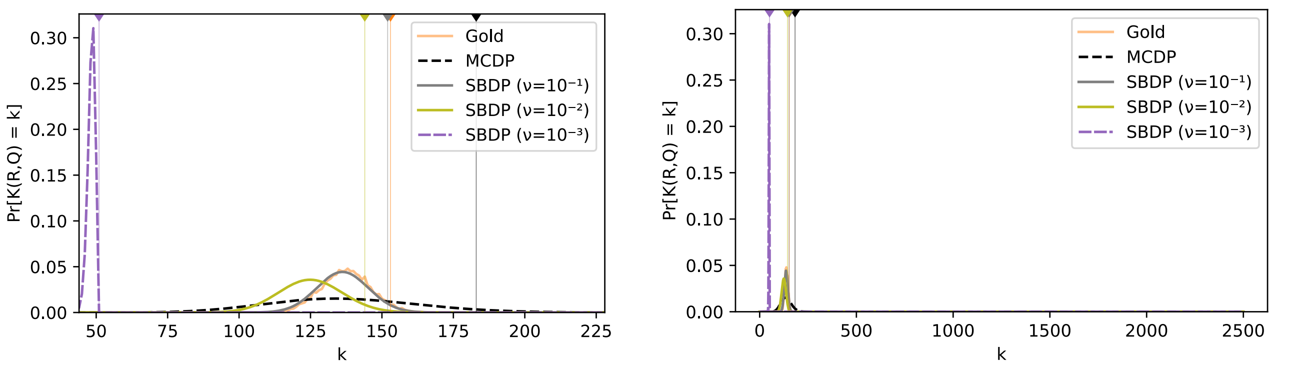


Figure S1: PMF of experiment from Table 4 with $|R| = 2,500$, $\ell_q = 1,000$, and $|Q| = 50$ (Line 26, second-to-last line in Table). This is a case where MCDP's critical value is more than 10% off. The right panel shows the full PMF and the left panel zooms in on the where most of the mass is. The left panel shows that MCDP's PMF is more heavy-tailed than the $\mathcal{H}_0^{GS}$ sampled PMF.

| annotation | n. intervals | avg. interval length | cov | $K(R,Q)$ | MCDP time (m) |
|---|---|---|---|---|---|
| CNV loss (inclusive) | 23,438 | 9,181 | 0.0695 | — | — |
| All genes | 217,527 | 348 | 0.0244 | 16,102 | 1,003 |
| Dominant (CGD) | 12,996 | 337 | 0.0014 | 764 | 4 |
| Recessive (CGD) | 21,950 | 295 | 0.0021 | 1,134 | 11 |
| Cancer | 8,289 | 351 | 0.0009 | 520 | 2 |
| DECIPHER | 680 | 386 | 0.0001 | 23 | $< 1$ |
| Essential | 44,183 | 329 | 0.0047 | 2,187 | 41 |
| GI1 | 23,438 | 9,181 | 0.0695 | 3,032 | 103 |
| HI1 | 52,712 | 341 | 0.0058 | 2,278 | 63 |
| ISCA | 4,748 | 399 | 0.0006 | 259 | 1 |
| No phenotype | 103,941 | 364 | 0.0122 | 8,233 | 218 |
| Non-coding | 10,005 | 383 | 0.0012 | 1,841 | 2 |
| OMIM | 48,709 | 314 | 0.0049 | 3,030 | 49 |
| Protein-coding | 209,158 | 347 | 0.0234 | 14,397 | 949 |

Table S1: Dataset statistics and running time for MCDP on data from Zarrei *et al.* (2015). The top row (CNV loss) corresponds to what was used as the reference annotation, and the rest of the rows correspond to the different query annotations. $K(R,Q)$ shows the size of the intersection between the reference and the query. The memory usage of MCDP was always under 370 MB and is not shown.

| Simulation parameters | | | | | Mean squared bias | | | | Divergence | | | |
|---|---|---|---|---|---|---|---|---|---|---|---|---|
| Reference | | Query | | | MCDP | SBDP($\nu$) | | | MCDP | SBDP($\nu$) | | |
| $\|R\|$ | cov | $l_q$ | $\|Q\|$ | cov | | $10^{-3}$ | $10^{-2}$ | $10^{-1}$ | | $10^{-3}$ | $10^{-2}$ | $10^{-1}$ |
| 50 | 0.005 | 10 | 500 | 0.005 | 0.004 | 5.430 | 0.066 | 0.070 | 0.001 | 603.264 | 0.010 | 0.011 |
| 50 | 0.005 | 10 | 5000 | 0.05 | 0.009 | 8.827 | 0.432 | 0.555 | 0.002 | 688.106 | 0.078 | 0.129 |
| 50 | 0.005 | 10 | 50000 | 0.5 | 0.000 | 0.000 | 0.001 | — | 0.000 | 0.567 | 0.158 | — |
| 50 | 0.005 | 100 | 50 | 0.005 | 0.002 | 0.626 | 0.078 | 0.007 | 0.000 | 1.265 | 0.095 | 0.001 |
| 50 | 0.005 | 100 | 500 | 0.05 | 0.007 | 4.916 | 0.763 | 0.070 | 0.001 | 313.083 | 0.976 | 0.006 |
| 50 | 0.005 | 100 | 5000 | 0.5 | 0.009 | 2.922 | 4.983 | 0.430 | 0.001 | 688.299 | 10.775 | 0.116 |
| 50 | 0.005 | 1000 | 5 | 0.005 | 0.002 | 0.009 | 0.008 | 0.002 | 0.002 | 0.003 | 0.001 | 0.000 |
| 50 | 0.005 | 1000 | 50 | 0.05 | 0.007 | 0.084 | 0.082 | 0.012 | 0.003 | 0.016 | 0.014 | 0.001 |
| 50 | 0.005 | 1000 | 500 | 0.5 | 0.048 | 0.788 | 0.736 | 0.069 | 0.003 | 0.263 | 0.222 | 0.003 |
| 500 | 0.05 | 10 | 500 | 0.005 | 0.008 | 54.258 | 0.675 | 0.697 | 0.002 | 687.771 | 0.102 | 0.109 |
| 500 | 0.05 | 10 | 5000 | 0.05 | 0.033 | 88.187 | 4.392 | — | 0.005 | 686.977 | 0.832 | — |
| 500 | 0.05 | 10 | 50000 | 0.5 | 0.000 | 0.002 | — | — | 0.000 | 5.054 | — | — |
| 500 | 0.05 | 100 | 50 | 0.005 | 0.007 | 4.915 | 0.781 | 0.071 | 0.005 | 307.615 | 1.017 | 0.006 |
| 500 | 0.05 | 100 | 500 | 0.05 | 0.017 | 49.161 | 7.619 | 0.686 | 0.006 | 687.505 | 10.315 | 0.061 |
| 500 | 0.05 | 100 | 5000 | 0.5 | 0.036 | 29.212 | 49.841 | — | 0.004 | 687.223 | 112.519 | — |
| 500 | 0.05 | 1000 | 5 | 0.005 | 0.007 | 0.219 | 0.076 | 0.008 | 0.111 | 35.211 | 0.013 | 0.001 |
| 500 | 0.05 | 1000 | 50 | 0.05 | 0.011 | 2.210 | 0.779 | 0.068 | 0.107 | 2.224 | 0.133 | 0.003 |
| 500 | 0.05 | 1000 | 500 | 0.5 | 0.062 | 21.740 | 7.417 | 0.640 | 0.047 | 33.173 | 2.301 | 0.023 |
| 2500 | 0.25 | 10 | 500 | 0.005 | 0.034 | 110.321 | 3.284 | — | 0.026 | 687.094 | 0.606 | — |
| 2500 | 0.25 | 10 | 5000 | 0.05 | 0.078 | 441.283 | — | — | 0.022 | 686.271 | — | — |
| 2500 | 0.25 | 10 | 50000 | 0.5 | 0.001 | — | — | — | 0.000 | — | — | — |
| 2500 | 0.25 | 100 | 50 | 0.005 | 0.014 | 7.377 | 3.908 | 0.359 | 0.093 | 687.627 | 7.098 | 0.042 |
| 2500 | 0.25 | 100 | 500 | 0.05 | 0.036 | 74.747 | 38.164 | — | 0.089 | 686.872 | 71.399 | — |
| 2500 | 0.25 | 100 | 5000 | 0.5 | 0.040 | 146.131 | — | — | 0.028 | 686.520 | — | — |
| 2500 | 0.25 | 1000 | 5 | 0.005 | 0.012 | 2.821 | 0.393 | 0.038 | 0.714 | 687.719 | 0.120 | 0.003 |
| 2500 | 0.25 | 1000 | 50 | 0.05 | 0.052 | 28.190 | 3.889 | 0.336 | 0.669 | 687.197 | 0.666 | 0.012 |
| 2500 | 0.25 | 1000 | 500 | 0.5 | 0.328 | 280.237 | 37.307 | — | 0.429 | 686.407 | 12.317 | — |

Table S2: Mean squared bias and average KL-divergence between an algorithm's and sampled $\mathcal{H}_0^{GS}$ overlap statistics probability mass functions. The experimental setup is identical to Table 4. The mean squared bias is the difference in mean values and is defined as $\frac{1}{t} \cdot \sqrt{\sum_{i=1}^{t} \left(\bar{K}_{\text{alg}}^{(i)} - \bar{K}_{\text{GS}}^{(i)}\right)^2}$, where $K_{\text{alg}}^{(i)}$ ($K_{\text{GS}}^{(i)}$) is the mean value of overlaps statistics calculated from the probability mass function, estimated by an algorithm (sampling under $\mathcal{H}_0^{GS}$) for $i^{\text{th}}$ replicate out of $t = 10$ sampled annotations.

| dataset | Sampled $\mathcal{H}_0^{GS}$ | MCDP | SBDP ($\nu = 10^4$) | SBDP ($\nu = 10^3$) |
|---|---|---|---|---|
| *EC* | 44 | 42 | 40 | 40 |
| *CS* | 269 | 271 | 516 | 249 |
| *CNV* | 674 | 665 | 701 | 688 |
| *H3K4me3* | 1,956 | 1,961 | 2,050 | 1,864 |

Table S3: Critical values at significance level of 0.05 for the real datasets from Sarmashghi and Bafna (2019).

| Label | Description |
|---|---|
| All | all human genes (RefSeq) |
| Dominant/Recessive | curated lists of genes by Clinical Genomic Database (CGD) (`https://research.nhgri.nih.gov/CGD/`) |
| Cancer | curated list of genes by Cancer Gene Census (`http://cancer.sanger.ac.uk/cosmic/census`) |
| DECIPHER | curated list of genes associated with pathologies of 70 specified syndromes (`https://decipher.sanger.ac.uk/syndromes`) |
| Essential | human orthologs of sixs mouse phenotypes, considered to be essential genes, downloaded (from Mouse Genome Informatics) |
| GI1 | genes with residual variation intolerance score $GI \leq -0.5381322$, predicted by Petrovski *et al.* (2013) |
| HI1 | genes with either haploinsufficiency score $HI > 0.4$, predicted by Huang *et al.* (2010) |
| ISCA | Dosage sensitive genes by ISCA (`http://www.ncbi.nlm.nih.gov/projects/dbvar/ISCA`) |
| No phenotype | Genes where a mutation (SNP, CNV) does not lead to a defined phenotype (manually done by Zarrei *et al.* (2015)) |
| OMIM | genes associated with diseases (`ftp://ftp.ncbi.nih.gov/repository/OMIM/morbidmap`) |

Table S4: Short description of gene groups used in Figure 4; the full description can be found in the supplement of Zarrei *et al.* (2015). The list of gene names used by Zarrei *et al.* (2015) is available in their supplement. We attempted to reconstruct the original dataset by downloading the coordinates of all the genes from UCSC RefSeq (track `ncbiRefSeq` of human genome hg19). In the case that there are multiple entries with the same gene name, we used all the entries. We could not locate around 3,000 gene names in RefSeq; those were excluded from our groups. The final list of genes and the coordinates of each gene set is available in our reproducibility repository

# References


Graham, R. L. *et al.* (1994). *Concrete Mathematics: A Foundation for Computer Science*. A foundation for computer science. Addison-Wesley.

Huang, N. *et al.* (2010). Characterising and predicting haploinsufficiency in the human genome. *PLoS genetics*, **6**(10), e1001154.

Petrovski, S. *et al.* (2013). Genic intolerance to functional variation and the interpretation of personal genomes. *PLoS genetics*, **9**(8), e1003709.

Sarmashghi, S. and Bafna, V. (2019). Computing the Statistical Significance of Overlap between Genome Annotations with ISTAT. *Cell Systems*, **8**(6), 523–529.

Zarrei, M. *et al.* (2015). A copy number variation map of the human genome. *Nature Reviews Genetics*, **16**(3), 172–183.
